# Effects of Different Finger Grips and Arm Positions on the Performance of Manipulating the Chinese Brush in Chinese Adolescents

**DOI:** 10.3390/ijerph181910291

**Published:** 2021-09-29

**Authors:** Ching-Hung Lee, Yu-Chi Lee

**Affiliations:** 1 School of Public Policy and Administration, Xi’an Jiaotong University, Xi’an 710049, China; leechinghung@xjtu.edu.cn; 2 School of Design, South China University of Technology, Guangzhou 510006, China

**Keywords:** Chinese calligraphy handwriting (CCH), finger grip, wrist position, hand–eye coordination, Chinese brush, hand tools evaluation, finger motors

## Abstract

This study aimed to investigate the effects of finger grip and wrist position on Chinese calligraphy handwriting (CCH). Thirty participants were recruited in the study and asked to manipulate the Chinese brush using two finger grip methods (three-finger grip and five-finger grip) and two wrist positions (suspended wrist and raised wrist). Three experimental writing tasks were applied to investigate writing stability, agility, and hand–eye coordination, and to evaluate the completion time (s), area of error (cm^2^), and error times. Subjective responses (arm aching level, ease of grip, exertion level, and comfort) regarding the four combinations of Chinese brush manipulation were measured. The results indicated significantly better performance with the three-finger grip for the stability and agility tests, and with the five-finger grip for the hand–eye coordination task. Using the suspended wrist position for CCH allowed better agility and hand–eye coordination than the raised wrist position. In consideration of the results of the four operational combinations, the three-finger grip with a suspended wrist position demonstrated the best performance in both objective and subjective measurements. It is recommended for application in the early learning stage. These findings can be considered when teaching Chinese brushes for beginners of CCH in schools.

## 1. Introduction

Chinese calligraphy handwriting (CCH) is defined as the process of using a Chinese brush to write characters [1,2]. It is a process that involves three-dimensional hand movements with a brush on paper. CCH is a high-end artistic performance and activity that places great demands on the mind and requires sufficient mental abilities. CCH is also culturally relevant to the Chinese population, especially among the elderly and adults [3,4]. In addition, CCH training is helpful for developing digital motor control in children [5,6]. Due to these benefits, introductory calligraphy courses are included in most primary and secondary school curricula in China and Japan. There are also specialized calligraphy programs and courses at senior schools for the elderly.

Applying CCH in training induces positive results in several parts of the human body. The benefits of CCH as mind–body exercises for the improvement of mental health [2,7], mild cognitive impairment [8], emotional stability in cancer survivors [9], spatial working memory [10], stress control in individuals [11], and attention control [12] have been reported. Moreover, Chen et al. [13] emphasized that practicing CCH had a significant effect on improving brain network efficiency. In particular, the long-term effects of CCH training have a positive influence on brain network efficiency [14]. In recent decades, CCH research has mainly been conducted in the clinical, psychological, and rehabilitation fields. In previous studies, the manipulation of the Chinese brush was controlled in each experiment [9,10,11]. The differences caused by different Chinese brush manipulations require more attention. Furthermore, few studies have used quantitative and experimental methods to evaluate the performance of CCH from the perspective of ergonomics and in the context of Chinese brush calligraphy by focusing on the effects of different methods of manipulating the Chinese brush [12,13,14].

**Figure 1:**
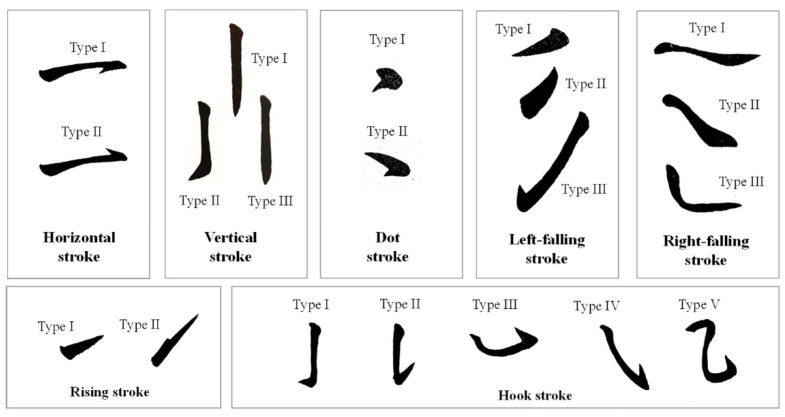
Illustration of calligraphy brushwork of the running script.

The importance and difficulties of CCH come from its intrinsic relationship with calligraphy scripts, brush strokes, and calligraphy brushwork [14,15]. Over three thousand years in ancient China, from the Warring States period and the Chin Dynasty (475–206 B.C.), the Han dynasty (206 B.C.–A.D. 220),and the Tang Dynasty (A.D. 618–A.D. 907), Chinese calligraphy evolved into five major calligraphy scripts, namely seal script, official script, regular script, running script, and cursive script (as shown in Appendix A). These extremely artistic writing methods have led to many calligraphy works by masters becoming treasures of Chinese culture [16,17,18]. Chinese calligraphy led the foundation for Chinese aesthetics and gave rise to Chinese ink painting, which greatly influenced Eastern aesthetics [14,15]. Broadly, there are seven types of brush “strokes” of CCH for the running script (Figure 1). These include horizontal, vertical, dot, left-falling, right-falling, rising, and hook strokes. These seven stroke types are dynamic. The running script requires a well-handled Chinese brush because the scripts and calligraphy brushwork are quite complicated in operation [14]. In general, different strokes in CCH are performed by manipulating the Chinese brush differently based on the user’s experiences and preferences. Many studies have shown that the correct operational method is the main factor affecting work performance and subjective feelings, regardless of the specific hand tool used [19,20]. Therefore, the best method of holding the brush and using it well is a critical and fundamental issue. This issue is also the motivation and value of this study.

The Chinese brush can be manipulated by combining a finger grip with a wrist position. In practice, there are many different finger grips, such as applying two fingers to pinch the brush handle, or placing four fingers together vertically on the side of the brush handle with the thumb pressing forward from the back. For general users, the three-finger grip and five-finger grip are the most popular and commonly used. The wrist position can be divided into four types: rested, cushioned, suspended, and raised. Thus, by combining different wrist positions and finger grips, numerous Chinese brush manipulation combinations can be produced. At present, a consensus on the best way to manipulate the Chinese brush for beginners of CCH has not yet been investigated.

When performing CCH, there are demanding requirements, including hand stability, agility, and hand–eye coordination, when using the Chinese brush. Using the correct operating combination in the early learning stage can improve performance, enhance the ease of learning, and promote skill development [14]. However, information on the recommended Chinese brush-holding posture is still lacking. Hence, this study aimed to conduct quantitative evaluations to investigate the effects of different finger grip and wrist positions on CCH performance. The CCH performance was measured using three track tasks designed to assess motor skills in terms of stability, agility, and hand–eye coordination. Subjective ratings were recorded for each method of Chinese brush manipulation. The goal of this study was to identify which Chinese brush manipulation method was the best method for performing CCH. The findings can be applied to benefit users practicing CCH in the early stages.

## 2. Materials and Methods

### 2.1. Participants

In this study, 30 undergraduate and graduate students (15 males and 15 females), aged between 18 and 23 years, were recruited. The mean age and hand length of the participants were 21.7 ± 5.7 years and 16.2 ± 1.3 cm, respectively. All participants were right-handed. Self-reports indicated no history of hand injuries or vision dysfunction. None of the participants had more than a few months of basic CCH training; hence, all the participants were considered beginners in performing CCH [13]. Each participant was adequately informed about the purpose and procedures of this study and signed an informed consent form before the experiment. The study protocol was approved by the Institutional Ethics Committee.

### 2.2. Experimental Apparatus

The Chinese brushes used in this study are commonly used in China and are easily available from a bookstore. The length of the Chinese brush was 260 mm, with a 30 mm length of brush hair. The diameter of the handle was 10 mm. For professionals, the brush hair material directly impacts CCH performance, as a certain kind of hair material is considered more appropriate for a certain script style. Hair size (large, medium, small) and texture (soft, mixed, hard) were also selected for particular writing styles. To control these variations of the Chinese brush and fit the beginner user’s demands, the Chinese brush used in this study was made of a mix of goat and Siberian weasel hair of medium size. The beginners were required to manipulate these medium-size brushes to practice and produce lines of different thicknesses. Medium-sized brushes are the most widely used. A 230 mm rounded bamboo was used as the Chinese brush holder. The holder’s surface was smooth and without a groove. Moreover, commercially available ink, inkstones, and rice paper (210 × 297 mm) were provided by an experimenter to avoid the influence of these variables on writing performance. A stopwatch (Jadco, Stopwatch, Victoria, Australia) with a resolution of 0.01 s was used by an experimenter to measure the time taken to complete each experimental task.

### 2.3. Operating Methods for the Chinese Brush

**Figure 2:**
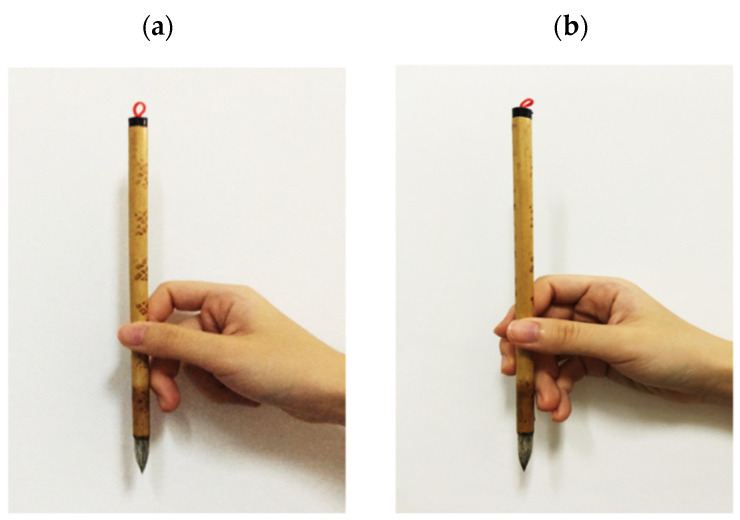
The two typical finger grips for manipulating the Chinese brush in the study. (**a**) Three-finger grip; (**b**) Five-finger grip.

Owing to the various brush strokes performed for particular purposes, there are many different methods for manipulating the Chinese brush. Using the finger grip to manipulate the Chinese brush correctly is the most basic and important consideration for beginners when learning CCH [14]. Hence, four common methods for manipulating Chinese brushes were evaluated in this study. The manipulation method can be divided into two major factors: finger grip and wrist position. There are two typical finger grips for beginners: the three-finger grip and the five-finger grip (as shown in Figure 2).

**Figure 3:**
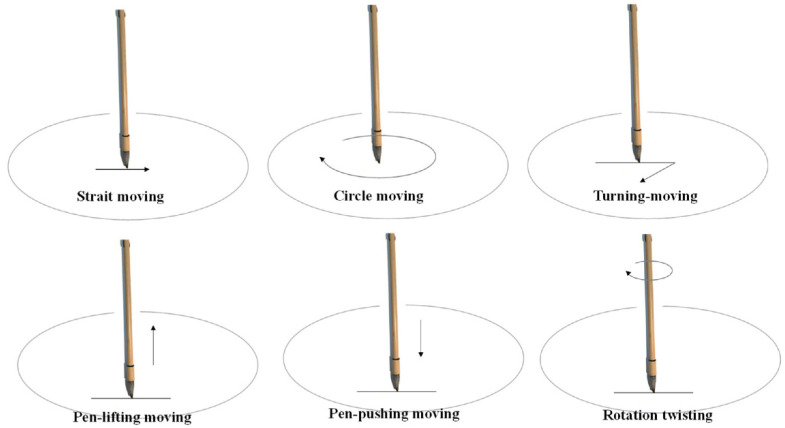
Illustration of six brushwork types.

CCH the process of the three-dimensional movement of the brush on the paper by the hand, which can also be said to be the movement of the penholder. Calligraphy brushwork is an essential component of calligraphy. There are six brushwork types: “straight moving,” “circular moving,” “turning moving,” “pen-lifting moving,” “pen-pushing moving,” and “rotating twisting” (as shown in Figure 3) [15,21]. Advanced handwriting skills require a better finger grip with a suitable wrist position to skillfully control the Chinese brush. The stroke depth refers to the reduction in height after pushing the pen tip to the paper surface during the writing process, denoted by h. There is a linear relationship between handwriting width and stroke depth, and the functional relationship between handwriting width and stroke depth can be expressed as w = f(h) (Appendix B). Thus, pen-lifting and pen-pushing movements can control the shift of the stroke line [15,21].

The thumb, middle, and index fingers, named Tankouhou in Japan, are used for the three-finger grip [13,14]. The Chinese brush is held vertically and gripped straight using the thumb and index finger. The middle finger lightly contacts the handle on the lateral side. Keeping the handle rested on the middle finger maintains the balance between the three coordinative fingers. The ring and little fingers curl tightly and press against the base of the palm, with no contact with the Chinese brush. The user is requested to imagine an egg-shaped space inside the palm when gripping a Chinese brush. When performing CCH, the user is asked to hold the Chinese brush pointed downward on the rice paper at a right angle (90° to the rice paper).

For the five-finger grip, which is called Soukouhou in Japan, the brush needs to be held with all five fingers [13,14]. The thumb, index, and middle fingers were used to hold the brush firmly and keep the handle straight. Additionally, the Chinese brush is held between the middle and ring fingers to maintain balance. The little finger closes with the ring finger for support. The palm is shaped as if hiding an egg, meaning that there is an empty space inside the palm. The user needs to keep the wrist and palm loose and at a smooth angle to avoid fatigue, regardless of the finger grip. The holding position of the thumb and index fingers is approximately two-thirds of that along the handle from the top.

**Figure 4:**
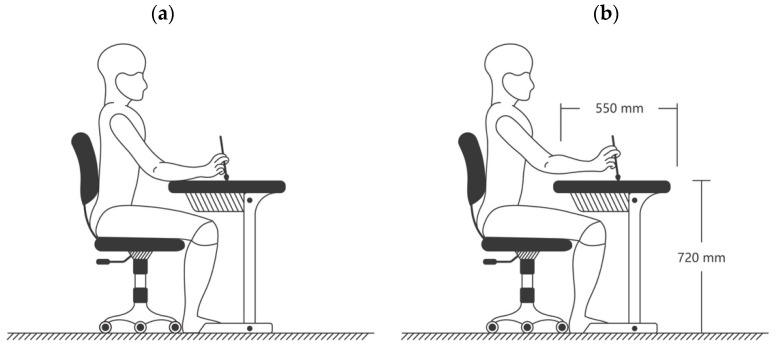
The experimental setting with a demonstration of the (**a**) suspended wrist and (**b**) raised wrist position.

Based on various writing purposes, different wrist positions were selected for diverse CCH styles. The common wrist positions for manipulating the Chinese brush are as follows: rest, cushion, suspended wrist, and raised wrist. Resting the wrist and forearm on the table enables the use of the fingers. The rested wrist position is good for writing small or extremely small characters. The cushioned wrist position is defined as resting the dominant wrist on the surface of the non-dominant wrist on the table. This wrist position is used to write ordinary small characters. In the suspended wrist position, the user keeps the elbow and forearm on the table comfortably, and the user is asked to lift the wrist slightly from the table for writing. When using a raised wrist position, the user is requested to raise the elbow, forearm, and wrist without touching the table during writing. In general, the user needs to lift his or her forearm 5 cm away from the table when using the raised wrist position. Since it is not appropriate for beginners to practice writing small characters at first, the rest and cushion wrist positions were not used in the present study. The suspended wrist position is commonly used to practice medium characters [13,14]. For users who intend to enhance their CCH skills to the level of art, a raised wrist position is recommended from the very beginning [14]. Thus, the suspended wrist and raised wrist positions (see Figure 4) for manipulating the Chinese brush were selected in this study to compare the differences in CCH performance.

### 2.4. Writing Performance Indicators

**Figure 5:**
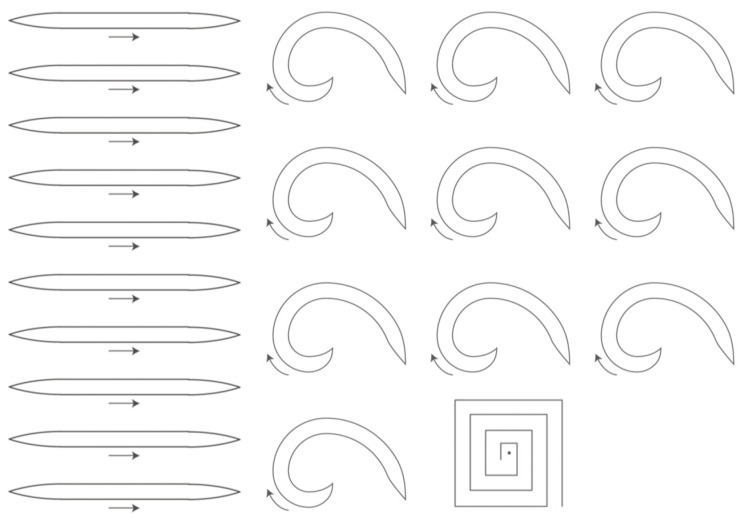
Three writing tasks (straight, curved, and maze tracks) used in this study.

As in the above-mentioned illustration of six brushwork types (Figure 3), using a Chinese brush to write characters presents difficulty in controlling the three-dimensional movement. Thus, we chose a simple and fundamental brushwork movement for a beginner as our experimental writing task. To investigate the effects of the finger grip and wrist position on calligraphy writing performance, the experiment consisted of three writing tasks (straight, curved, and maze tracks) (see Figure 5). Performance in the three tasks was recorded and used to assess the motor skills of stability, agility, and hand–eye coordination. These three abilities are important for CCH, particularly for controlling the brush.

#### 2.4.1. Straight-Track Task

Each participant was asked to sit on an adjustable seat to write along the tracks under different combinations of finger grip and wrist positions to manipulate the Chinese brush. In the straight-track task, the participants were required to perform 10 straight tracks one by one. Each track was written in one continuous movement—writing in segments was not allowed. When a participant performed the 10 tracks successfully, the stopwatch was stopped, and the indicated time was recorded as the completion time. A computer was used to calculate the failure area among the 10 tracks. The straight-track task was repeated twice, and the average of the data was used in the subsequent analysis. The path length and breadth of each straight track were 50 and 6 mm, respectively. To achieve this task, we also provided an operational definition of stability. To execute the task, the participants needed to press down the Chinese brush, move along the straight line stably, and release the brush at the end to fill the straight track. This is a basic CCH stroke. Therefore, the straight-track task was used as an indicator to evaluate the motor skill of stability.

#### 2.4.2. Curved-Track Task

An adjustable seat was provided to the participant while sitting before the experiment. Participants were asked to adjust their seat to a comfortable height. The setting of the curved-track task was similar to that of the straight-track task. The curved tracks of 6 and 50 mm total length were modified from the Fibonacci helix and applied to perform the CCH stroke in this study. Ten curved tracks were printed on a piece of rice paper. The participants were requested to perform all 10 curved tracks individually and repeat the task twice under each selected Chinese brush condition. To achieve this task, we also provided an operational definition of agility. In the task, participants adjusted the direction constantly by rotating the handle and moving the wrist to control the movement of the Chinese brush according to the curved track. Participants were required to ensure that the Chinese brush rotated while the wrists flexed to complete the task. The skills required good agility to perform basic movements in CCH. Thus, the average completion time and area of error were measured to evaluate agility under different combinations of finger grips and wrist positions when manipulating the Chinese brush.

#### 2.4.3. Maze Track Task

The participants were asked to perform the maze track task while they sat on a chair at their preferred height. Each participant was required to repeat the maze track task twice. The characteristics of the maze track were designed with a path length of 300 mm and a width of 6 mm between each line. At the beginning of the task, participants were instructed to start at the black dot and use the same effort as they would for normal writing. Participants needed to draw a line continuously along the spiral maze in an inside-out manner to reach the exit of the maze, as if performing CCH normally. The maze track combined various directions, so hand and eye coordination was required to complete the task, which included direction changes and movements. The task was designed with a square-shaped Archimedean spiral maze adapted from the work of Goonetilleke et al. [22] and Cheng et al. [23]. The maze task is commonly used to determine hand–eye coordination [24,25]. To achieve this task, we also provided an operational definition of hand–eye coordination. Hand–eye coordination is a visual system capability that coordinates the revised information to control the hands to complete a task [26,27], such as writing [24]. Therefore, the maze track task was selected to evaluate the participants’ hand–eye coordination by measuring the completion time and errors in different ways of manipulating the Chinese brush. Errors were defined as the number of times the handwriting crossed the edges of the maze. Drawing discontinuously was considered an error. A shorter completion time and fewer errors were referred to as better performance in the task.

During the straight-track and curved-track tasks, participants drew the printed tracks on the rice paper as closely as possible. The writing areas that exceeded the track contour and those that did not fill the track contour were defined as areas of error. The average area of error between repeat trials was used to evaluate the participant’s motor control ability. The smaller the failure area, the better the performance of controlling the skills required for manipulating the Chinese brush. When participants completed all the tasks, the rice papers were carefully collected. All rice papers were left for 30 min to allow the ink to dry. Subsequently, the rice paper with dried ink was scanned to a digital image (resolution: 300 dpi). The images were exported to a PC and processed using Adobe Photoshop to measure the error area. The ink outlines were carefully selected using the pen tool function. Subsequently, the selected ink image was overlapped with the original design of a straight or curved track. The software automatically calculated the mismatch area (overfilled/unfilled), and the unit of area of error was recorded in cm^2^. One experimenter was assigned to calculate all areas of error to eliminate potential inter-examiner errors.

### 2.5. Experimental Procedure

A 1000 mm (width) × 550 mm (depth) × 720 mm (height) experimental table was provided in the office. Each participant was taught two different finger grips (three-finger and five-finger) and two wrist positions (suspended wrist and raised wrist) by an experimenter. Before collecting the experimental data, the participants were asked to practice manipulating the Chinese brush in different operating combinations to familiarize themselves. The four combinations for manipulating the Chinese brush were as follows: (1) three-finger grip with a suspended wrist (TS), (2) three-finger grip with a raised wrist (TR), (3) five-finger grip with a suspended wrist (FS), and (4) five-finger grip with a raised wrist (FR). To evaluate the effects on writing performance, Chinese brush manipulation was randomly assigned. The participants completed the three writing tasks (straight track, curved track, and maze track) for each combination and repeated each task twice. The average completion time of each task was recorded. The order of the three tasks was randomized for each participant. To minimize fatigue, each participant took a 5 min break between trials.

When each participant completed the three track tasks using a specific operation, the participant was asked to respond to a semantic questionnaire that contained questions about the arm aching level, ease of grip, exertion level, and comfort. A score of 1 to 9 was used to rate each subjective feeling. The phrases “arm not aching, grip easily, exertion easily, and comfortable” were rated as scores of 9. The previous semantic-differential scale questionnaire for evaluating the motor skill performance of the fingers was applied in this study [28,29,30].

### 2.6. Data Analysis

The experimental data collected in this study were analyzed using SPSS (version 22.0; SPSS Inc., Chicago, IL, USA), with the significance level set at 0.05. The independent variables were finger grip and wrist position. The dependent variables were the completion time of each task, areas of error in straight- and curved-track tasks, and errors in the maze track task. The above measurements were used to evaluate the motor ability of stability, agility, and hand–eye coordination when using different combinations to manipulate the Chinese brush. A two-way analysis of variance (ANOVA) was performed to evaluate the relationship between the independent variables and the measured indices. In addition, a one-way ANOVA was applied to determine the optimal combination of finger grip and wrist position for CCH. The subjective responses were also used to evaluate which combination produced a better feeling under each of the Chinese brush operations. Duncan’s multiple range test (MRT) was conducted for post hoc comparison. Before the data analysis, all the measures verified the normality and homogeneity of variances using the Kolmogorov–Smirnov test and Levene’s test, respectively. The results indicated that the data collected in this study were normally distributed and homogenous. Hence, ANOVA was performed.

## 3. Results

The results of the two-way ANOVA are summarized in Table 1. Values are presented as mean ± standard deviation. For the main effect of finger grip, significant differences were observed in the area of error for stability (*p* < 0.001) and agility (*p* = 0.003) and in the indicators of errors for hand–eye coordination ability (*p* = 0.025). No significant difference was found in the measure of completion time. For the main effect of wrist position on CCH performances, the results indicated that wrist positions significantly influenced the area of error in agility (*p* = 0.017) and errors in hand–eye coordination (*p* = 0.031). Additionally, the ANOVA results revealed a significant interaction between the finger grip and wrist position on all selected measurements, except for the completion time for the stability task (*p* = 0.093).

To further investigate the differences in the combinations for manipulating the Chinese brush, a one-way ANOVA was conducted. The results are summarized in Table 2. In measurements of completion time, a significant difference between the four combinations was found in the agility task. Duncan’s MRT results indicated that the FS method had the worst performance (90.52 s) in the agility task. In addition, significant differences among the four combinations were found in the stability and agility tasks in the area of error measurements. Applying the FR operation to the Chinese brush manipulation resulted in the worst stability (166.66 cm^2^) and agility (259.86 cm^2^) of all the operations based on the results of Duncan’s MRT results. In hand–eye coordination ability, the ANOVA results showed that there was a significant effect on error measurement. According to Duncan’s MRT results, using a TR grip for manipulating the Chinese brush caused the most errors (5.93 times) in the hand–eye coordination test.

**Figure 6:**
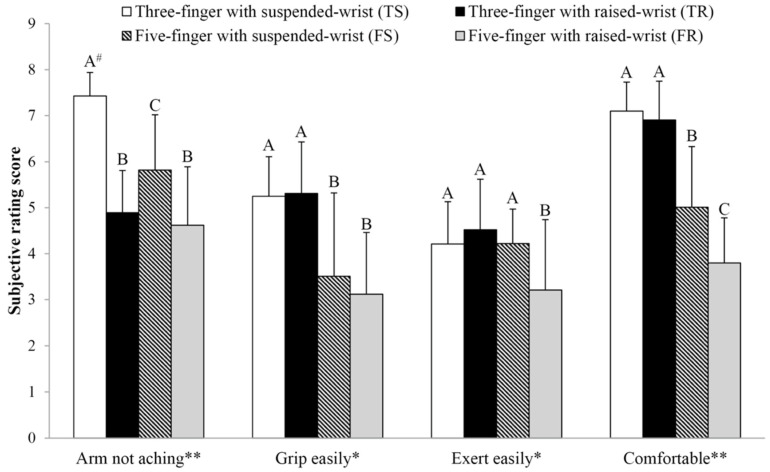
The differences between the four Chinese brush operations on selected subjective responses (*: *p* < 0.05; **: *p* < 0.01; ^#^ means post hoc group results, which means the A, B, C were significantly differed).

Figure 6 illustrates the ANOVA and Duncan’s MRT results for the subjective responses when manipulating the Chinese brush using the four combinations. Significant differences between the four Chinese brush operations were found for all the selected measures (all *p* < 0.05). The results showed that using the FR method led to the worst performance in all subjective measurements. Furthermore, the TS method was most appropriate for the participants in terms of the “arm not aching,” “ease of grip,” “exertion,” and “comfort” measurements.

## 4. Discussion

Chinese calligraphy is a classic traditional art in Chinese culture. Users apply different holding methods and wrist positions in response to different CCH performance needs. In the past, most of the research has investigated the effect of using Chinese brushes on brain development [13,31] and then applied the results to the fields of rehabilitation, education, and neuroscience [32,33,34]. Using the correct operation to manipulate the Chinese brush has been the main factor affecting CCH performance compared to other factors, including learning time and age of the participant [14]. There is a lack of available quantitative evaluations to study the differences in CCH performance using different writing operations. Therefore, the present study evaluated the effects of two typical finger grips and two wrist positions on stability, agility, and hand–eye coordination abilities when performing CCH. A most favorable combination of wrist position and grip for manipulation of the Chinese brush for practicing CCH was also determined.

The two-way ANOVA results showed that the type of finger grip had a significant effect on the performance in terms of the area and number of errors. The three-finger grip resulted in the least area of error in the straight- (114.57 cm^2^) and curved-track (205.03 cm^2^) tasks. This means that the three-finger grip for manipulating the Chinese brush has better stability and agility than the five-finger grip. When performing fine motor tasks or manipulating hand tools (such as brushes, chopsticks, screwdrivers, etc.), many fine hand muscles and nerves are involved in performing a complex operation. The effects of different finger grips for manipulating the Chinese brush during CCH are still uncertain, but previous studies reported that when operating hand tools, three fingers resulted in better speed, precision, and stability [5,29,30,35,36] compared to using five fingers. Similar results were observed in the present study. When manipulating the Chinese brush, the three-finger grip produced higher stability and agility during CCH performance than the five-finger grip. Using chopsticks and manipulating the Chinese brush may have similar difficulties, as both operations require a high level of fine motor skills in the fingers.

Furthermore, in the hand–eye coordination tasks, no significant difference was noted in the completion time between the two grip methods. The five-finger gripping method has significantly fewer errors (4.12 times) than the three-finger gripping method. Since the five-finger gripping method involves more fingers (middle, ring, and little fingers) to support the Chinese brush than the three-finger grip, it may enable the Chinese brush to maintain upright and press down more easily during CCH practice. Finger strength is known to change based on finger involvement during force production [37]. This implies that the participants could use more fingers for greater finger strength to control the Chinese brush when pressing down and touching the rice paper continuously to complete the complex movements and rotations in the maze track task. Previous studies have also revealed that using more fingers for a particular hand tool operation resulted in greater strength [28,31,38]. This may be the reason why the five-finger gripping method of manipulating the Chinese brush produced fewer errors than that of the three-finger gripping method in hand–eye coordination tasks.

In terms of the wrist positions, Table 1 indicates that the wrist position has a significant effect on the area of error in the agility task and the number of errors in the hand–eye coordination task. The results showed that the use of the suspended wrist position performed better than the raised wrist position. In this study, the raised wrist position requires the user to raise their forearm and wrist 5 cm away from the desktop and keep no contact with the desktop when performing CCH. Richards et al. [39] reported that the forearm position affects exertion. Holding an extremity away from the body (e.g., raised arm) would likely cause shaking [40] and lead to poor performance in operating hand tools [41]. The results of the study showed that relatively poor subjective ratings were found when using the raised wrist position (see Figure 6), especially in the arm not aching. The results revealed that the participants perceived that the raised wrist position induced worse “arm aching” after practicing CCH. The subjective results consisted of the previous objective results and can be used as an explanation for the poor CCH performance results of the raised wrist position in the study. The raised wrist position is an essential technique selected to train beginners who want to reach an artistic level of CCH performance [14]. The findings of this study highlighted that asking beginners to use the raised wrist position for CCH was difficult and uncomfortable. Since this study mainly evaluated the results of short-term effects, after a longer period of practice, arm aching after CCH performance may be improved.

In addition, the results of this study demonstrated that the interaction between the finger grip and wrist position significantly influenced the majority of selected indicators, except for the completion time of the stability task (as shown in Table 1). Furthermore, this study compared the differences among the four combinations of Chinese brush operations to determine the optimal operation. The comparison results in Table 2 show that the FR method performed the worst in the area of error in the stability and agility tests. The TR method produced more mistakes among the four different methods in the hand–eye coordination task, and the FS method had a longer completion time in the agility test than the other methods. Based on the objective results, the TS method led to better performance for all significant measurements. Moreover, Figure 6 shows that different combinations have significantly different effects on the subjective ratings. FR and FS have low subjective responses under the four selected indicators (“arm not aching,” “grip easily,” “exert easily,” and “comfortable”). Comparing the subjective assessments between the TS and TR methods, no significant difference was found, except in the “arm not aching” score (TS > TR). To summarize the above results, the TS method performed better in terms of stability, agility, and hand–eye coordination ability, and the TS method also generated significantly better subjective responses than the other Chinese brush operations. Considering both subjective and objective evaluation results, this study suggests that the TS method is a better method for practicing CCH than the other operational methods.

It is worth mentioning that completion time is a widely used indicator when evaluating new products, motor skills, and job performance [6,20,29,42,43,44,45,46]. Hence, the completion time was selected as an indicator to evaluate the difference in CCH performance under different operations in the study. Generally, the user is asked to perform CCH in an elegant posture and with a calm state of mind. The right training procedure in performing CCH can be beneficial to brain development, especially working memory [47]. Under this premise, the completion time of CCH may not be critical. In addition, to determine the human performance in different hand tool operations, product usability tests, and task evaluations, the use of area of error and rate of errors had good precision and efficiency [22,48,49,50,51]. This study involved the aforementioned indicators for evaluation. On the contrary, the completion time may be used as an index to evaluate professionalism and efficiency of performance in professional CCH populations. In professional calligraphers, faster completion time would mean better finger control, motor skills, and operational ability. The results of this study revealed that there was no significant difference in most of the completion time indicators. This may be because the participants recruited in this study were all beginners to CCH. Thus, using completion time to evaluate CCH performance may not be an efficient indicator for beginners and short-term practices. The relationship between completion time and CCH performance among beginners after a long period of practice warrants further investigation.

This study has a few limitations. In performing CCH, there are many kinds of finger gripping methods and wrist positions to deal with the specific writing needs of professionals. This study only compared the differences between the four common Chinese brush operations. The comparison of the remaining Chinese brush operations for a specific stroke within CCH performance can enrich the application. As mentioned before, only beginners were selected for evaluation. Better Chinese brush manipulating combinations for professional CCH artists also need to be confirmed in future research. Practice time is a critical factor that influences performance. Only short-term effects were evaluated in the current study due to time and resource constraints. The long-term effects of different methods of holding the Chinese brush deserve greater attention in future research. Multiple factors affect CCH performance. For example, the same height (720 mm) of the writing desk was applied to participants with various statures in the study. Providing an adjustable desk to fit individual needs could be considered a factor influencing writing performance. An analysis of CCH should determine the legibility, style, stroke, brushwork, scripts, and the correctness of character shapes.

## 5. Conclusions

This study quantitatively evaluated the effects of finger grip and wrist positions on CCH performance under the three selected measurements to validate the motor skills of stability, agility, and hand–eye coordination. The results indicate that finger grip and wrist position had a significant impact on performing CCH. The three-finger grip method generated better results than the five-finger grip method in terms of stability and agility. Fewer errors in the hand–eye coordination task were observed when using the five-finger grip. Applying the suspended wrist position resulted in better agility and hand–eye coordination during CCH performance than that of the raised wrist position. Additionally, the results of this study reported that the use of different Chinese brush operations affected CCH performance. From the objective measurements and subjective rating results, the TS manipulation resulted in a better CCH performance than the other combinations. Overall, the findings of this study recommend that the TS operation be used to manipulate the Chinese brush when practicing CCH. The findings of this study can provide useful information for the development of a gripping auxiliary device for Chinese brushes and guidance for novices during CCH practices.

Since previous studies still have no consensus on the best suggested method for using the Chinese brush for beginners, the major contribution and novelty of the current study was applying human factor-based evaluation of hand tools (Chinese brush) by measuring motor skills (stability, agility, and hand–eye coordination) to evaluate CCH performance. Additionally, the users’ subjective and objective responses were both considered for the determination. Eventually, the better combination of grip method and wrist position for performing CCH was the three-finger grip with a suspended wrist position. The findings can provide helpful information for beginners to enhance CCH while learning Chinese calligraphy.

Based on these findings and contributions, future research directions are based on two aspects. On the one hand, more writing tasks could be designed together with experts due to the practical brushwork types, i.e., “strait moving,” “circular moving,” “turning moving,” “pen-lifting moving,” “pen-pushing moving,” and “rotating twisting.” On the other hand, more complicated and classic calligraphy pieces could be used as writing performance indicators for experienced learners. Moreover, more brush strokes and brushwork should also be further extended to the experimental task design. These two research directions can further enhance our capability of helping Chinese calligraphy learners toward high-level Eastern aesthetics.

## Figures and Tables

**Table 1:** Two-way ANOVA results of the finger grip and the wrist position on Chinese calligraphic handwriting performances.

Motor Abilities	Measures	Finger Grip (F)		Wrist Position (W)		Significance
		Three-Finger	Five-Finger	Suspended Wrist	Raised Wrist	
Stability	Completion time (s)	57.72 ± 23.45 ^#^	59.69 ± 27.24	61.39 ± 26.81	56.07 ± 23.71	
	Area of error (cm^2^)	114.57 ± 50.00	147.17 ± 51.16	126.72 ± 56.39	135.13 ± 49.41	F ***, F × W ***
Agility	Completion time (s)	82.41 ± 33.59	83.16 ± 32.73	86.88 ± 32.56	78.66 ± 33.25	F × W *
	Area of error (cm^2^)	205.03 ± 73.22	233.69 ± 77.78	207.98 ± 74.96	239.95 ± 77.10	F **, W **, F × W **
Hand–eye Coordination	Completion time (s)	22.28 ± 8.68	21.63 ± 6.65	22.58 ± 7.08	21.30 ± 8.30	F × W *
	Errors (times)	5.06 ± 3.23	4.12 ± 3.19	4.14 ± 3.12	5.05 ± 3.31	F *, W *, F × W *

^#^: Mean ± standard deviation; *: *p* < 0.05; **: *p* < 0.01; ***: *p* < 0.001.

**Table 2:** The differences between the four Chinese brush operations on the stability, agility, and hand–eye coordination tests.

Motor Abilities	Measures	TS		TR		FS		FR		*p*-Value
Stability	Completion time (s)	57.61 ± 24.51		57.82 ± 22.56		65.23 ± 28.67		54.34 ± 24.85		0.122
	Area of error (cm^2^)	126.08 ± 56.19	A ^#^	123.06 ± 40.21	A	127.36 ± 57.07	A	166.66 ± 35.42	B	<0.001 ***
Agility	Completion time (s)	83.18 ± 33.57	A	81.65 ± 33.88	A	90.52 ± 31.43	B	79.67 ± 32.62	A	0.037 *
	Area of error (cm^2^)	208.02 ± 79.57	A	202.04 ± 66.82	A	207.95 ± 70.82	A	259.86 ± 76.32	B	<0.001 ***
Hand–eye Coordination	Completion time (s)	21.82 ± 6.36		22.74 ± 10.51		23.32 ± 7.70		19.91 ± 4.87		0.087
	Errors (times)	4.19 ± 2.78	A	5.93 ± 3.43	B	4.10 ± 3.44	A	4.15 ± 2.95	A	0.032 *

^#^: Duncan’s multiple range test results; *, *p* < 0.05; ***, *p* < 0.001; TS, three-finger with suspended wrist; TR, three-finger with raised wrist; FS, five-finger with suspended wrist; FR, five-finger with raised wrist.

**Figure A1:**
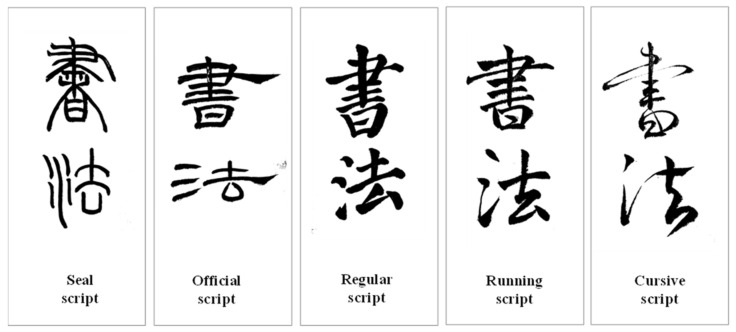
Five Major Calligraphy Scripts.

**Figure A2:**
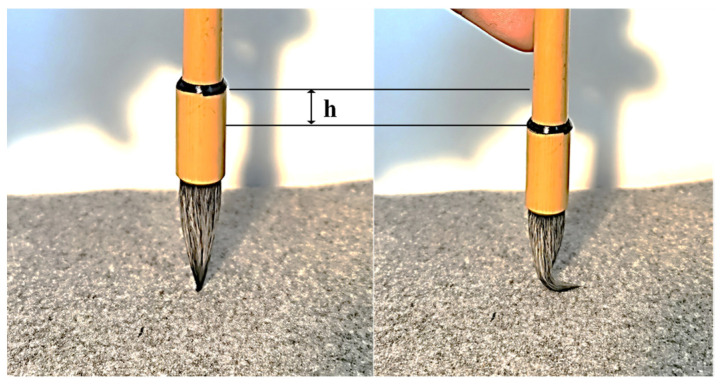
Original stroke depth (**left**) and writing stroke depth (**right**).

## Data Availability

The data presented in this study are available on request from the corresponding author. The data are not publicly available due to privacy reasons.
